# Validating a Numerical Simulation of the ConsiGma(R) Coater

**DOI:** 10.1208/s12249-020-01841-7

**Published:** 2020-11-26

**Authors:** Peter Boehling, Dalibor Jacevic, Frederik Detobel, James Holman, Laura Wareham, Matthew Metzger, Johannes G. Khinast

**Affiliations:** 1 Research Center Pharmaceutical Engineering GmbH, Graz, Austria; 2 GEA Process Engineering NV, Wommelgem, Belgium; 3 Merck & Co., Inc., Kenilworth, New Jersey USA; 4 IPPT, Graz University of Technology, Graz, Austria

**Keywords:** CFD-DEM, tablet coating, solid processes, heat and mass transfer

## Abstract

Continuous manufacturing is increasingly used in the pharmaceutical industry, as it promises to deliver better product quality while simultaneously increasing production flexibility. GEA developed a semi-continuous tablet coater which can be integrated into a continuous tableting line, accelerating the switch from traditional batch production to the continuous mode of operation. The latter offers certain advantages over batch production, *e.g.*, operational flexibility, increased process/product quality, and decreased cost. However, process understanding is the key element for process control. In this regard, computational tools can improve the fundamental understanding and process performance, especially those related to new processes, such as continuous tablet coating where process mechanics remain unclear. The discrete element method (DEM) and computational fluid dynamics (CFD) are two methods that allow transition from empirical process design to a mechanistic understanding of the individual process units. The developed coupling model allows to track the heat, mass, and momentum exchange between the tablet and fluid phase. The goal of this work was to develop and validate a high-fidelity CFD-DEM simulation model of the tablet coating process in the GEA ConsiGma® coater. After the model development, simulation results for the tablet movement, coating quality, and heat and mass transfer during the coating process were validated and compared to the experimental outcomes. The experimental and simulation results agreed well on all accounts measured, indicating that the model can be used in further studies to investigate the operating space of the continuous tablet coating process.

**Supplementary Information:**

The online version contains supplementary material available at 10.1208/s12249-020-01841-7.

## INTRODUCTION

Continuous manufacturing (CM) is increasingly applied in the pharmaceutical industry (1,2). In comparison to batch production, it enables a higher throughput while simultaneously decreasing the waste and energy consumption and production costs (3). Since the inline measurements are typically intrinsic to the process design, CM allows enhanced process control (4). Above all, CM requires a high level of process understanding (5,6).

In light of the transition from batch to continuous manufacturing, GEA developed the ConsiGma® continuous processing line to process powder raw materials into tablets (7). There are two types of ConsiGma® lines: the ConsiGma® continuous tableting line (CTL) and the continuous direct compression line (CDC). The advantages of both are a small footprint and reduced waste.

Tablets are the most commonly administered pharmaceutical dosage form. Tablets are often coated, *e.g.*, with cosmetic color coating, a protective layer (enteric or taste-masking) or a second active pharmaceutical ingredient, to differentiate products or to add functionality. All coating types have certain critical quality attributes (CQAs) that define the ultimate product performance. One of the most important CQAs are inter- and intra-coating variability (8), which should be low to ensure product performance and quality. Other CQAs are the appearance, water content and dissolution. Depending on the product the importance of the different CQAs can shift. Coating variability (CoV) is typically defined through experiments using samples taken during production. Uniformity can be assessed by comparing the weight of the film-coated tablet to the mean tablet core weight or *via* spectroscopy methods, such as terahertz or Raman probe. Also, Optical Coherence Tomography (OCT) is increasingly used as a novel real-time option (3,9).

Tablet coating experiments are performed on various scales (6,10,11), generally beginning with the smallest-scale equipment (laboratory scale) with only a few kilograms of tablets (10,12,13). Then, the laboratory equipment is typically scaled to the pilot scale with a drum load of 10–50 kg (14). Experiments on the industrial scale are carried out at the end of process development (15,16) using the intended commercial equipment and involving hundreds of kg of material. The product and technology transfer is accomplished based on existing empirical scale-up rules and experience of the operating engineers (14,17). However, scale-up often causes problems due to changes in the coater geometry designs across the scales or completely different drum designs on the production *vs.* the pilot scale. Thus, the general goal is to reduce the number of experiments required on a large scale, since they are expensive due to time and material consumption (16).

Although continuous tablet drum coaters are still rarely used in the pharmaceutical industry, the increasing interest in end-to-end continuous manufacturing has created the need for integrated continuous tablet coaters (18–20). Continuous tablet drum coaters typically have a similar shape and operating principles as traditional batch tablet coaters (18,21). The main difference is the design. For instance, in continuous tablet coaters, the baffles are constructed to direct the flow of the tablet bed from the inlet to the outlet region. The spray is applied from the top, and the drying airflow is either parallel or counter-current to the tablet movement. Such a fully continuous tablet coater, with an operating principle similar to the traditional batch pan coating process, may require more space and additional equipment.

GEA has developed a tablet coater that should eliminate these shortcomings and can be integrated into both ConsiGma® continuous production lines (6,22). It is a small semi-batch tablet coater, which can be nominally loaded with 2.5 to 7 kg of tablets depending on the drum insert. Compared to traditional batch tablet coaters, the ConsiGma® tablet coater operates at high rotation rates, with a Froude number close to 1. Streams of air (referred to as ‘air knives’) push the tablets away from the drum wall to form a cascade through the spray zone, allowing a very short coating cycle time (a total process time of about 10 min).

Simulations can be used to increase the understanding of the coating process. Especially, discrete element method (DEM) simulations can provide a detailed analysis of the coating process (23,24). Today it is possible to simulate the tablet coating process on every scale due to a relatively low number of tablets within the system (small batch size) and to analyze the process for several parameters (25). Simulations can offer information about the inter- and intra-tablet CoV, tablet velocity, tablet bed dynamics (*i.e.*, velocity, residence and cycle times) and forces acting on the tablets (17,26–28).

Computational Fluid Dynamics (CFD) has been increasingly used in industrial applications to simulate fluid flow (29,30). In recent years, CFD and DEM were coupled to model the interaction between granular and fluid materials. Initially, only the momentum exchange between fluid and solid phase was considered (29,31,32). Either only one phase was considered (one-way coupling) or both phases interacted with each other (two-way coupling). This allowed simulation of the movement of granular material, *e.g.*, in a conveyor or fluidized bed coater. However, in coating also mass and heat transfer between phases are important as coating involves also solvent drying. The exchange of mass and heat *via* a so-called four-way coupling (33) needs to be considered.

A full simulation of the ConsiGma® tablet coating process requires the above-mentioned four-way coupling approach. Although existing models do consider the heat and mass transfer from a macroscopic (integral) point of view, they do not resolve the local tablet state (*e.g.*, wetness or coating quality) (6,22). The approach presented in this work involves the development of an algorithm for the coupling of momentum, heat and mass transfer in the ConsiGma® film-coating equipment for realistically shaped tablets. A model for the fluid forces acting on the tablets was introduced that overcame the difficulty in modeling momentum transfer on a realistic tablet shape.

In summary, the goal of this work was to develop a model (a so-called *digital twin*) that simulates and predicts the entire coating process inside the ConsiGma® coater. This should allow testing of different operating conditions in terms of impact on the CQAs as well as optimizing the performance without conducting experiments.

The model itself was developed in three phases to isolate and validate the different phenomena occurring in the film coater. First, the tablet bed dynamics were validated by comparing experimental and simulation results for the tablet acceleration. Next, the coefficient of inter-tablet CoV was compared between model and experiments. Finally, the heat and mass transfer in the simulation was compared to experimental results. To that end, the outlet air temperature/humidity and the tablet moisture (measured *via* the loss on drying (LoD)) and temperature were analyzed. The goal of the validation was to prove that our fully mechanistic digital twin can be used for the design, control, and optimization of the ConsiGma® coater.

## Simulation Model

The CFD-DEM simulation model is introduced in the following section. It is followed by an explanation of the biconvex model development and concludes with a detailed presentation of the momentum, heat, and mass transfer model.

### CFD-DEM Model

The code XPS (eXtended Particle System) running on Graphics Process Units (GPUs) was used to calculate the particle motion (34). It solves Newton’s second law by accounting for particle-particle and particle-wall interactions (35,36). This method was developed by Cundall and Strack (37) in 1979. For the motion of each particle, the following equation is solved:

1 $$ {m}_P\frac{d{\mathbf{v}}_{\boldsymbol{P}}}{dt}=-{V}_i\nabla \mathrm{p}+{\overrightarrow{\boldsymbol{F}}}_i^d+\sum \limits_0^{N_P}{\mathbf{F}}_{P\to P}+\sum \limits_0^{N_w}{\mathbf{F}}_{P\to W}+{m}_p\mathbf{g} $$

where *m*_*P*_ is the particle mass, **v**_P_ is the particle velocity, −*V*_*i*_ ∇ *p* is the pressure gradient force, ${\overrightarrow{\boldsymbol{F}}}_i^d$ is the drag force, **F**_P->P_ is the particle-particle force, **F**_P->W_ is the particle-wall force and g is the gravity constant. The three-dimensional angular momentum of the particle is calculated as:

2 $$ {\mathbf{I}}_P\frac{d{\boldsymbol{\upomega}}_{\mathrm{P}}}{dt}=\sum \limits_0^{N_c}{\mathbf{M}}_P+{\mathbf{M}}_W+{\mathbf{M}}_F $$

where **ω**_P_ is the angular velocity, **M**_P_ is the torque induced by other particles, **M**_w_ is the torque due to tablet-wall interactions, **M**_F_ is the torque generated by the surrounding fluid, and **I**_P_ is the moment of inertia. Details of the algorithm can be found in (34) and (38)**.**

To simulate the gas phase, a commercially available CFD code (AVL Fire©) was used, which models the gas phase by solving the volume-averaged Navier-Stokes equations. All variables are locally volume-averaged quantities over a control volume V, which must be at least one order of magnitude larger than the particle volume V_P_ (38)**.** The conservation of mass is given by

3 $$ \frac{\partial }{\partial t}\left({\varepsilon}_F\cdot {\rho}_F\right)+\nabla \cdot \left({\varepsilon}_F\cdot {\rho}_F\cdot {\mathbf{v}}_{\boldsymbol{F}}\right)=0 $$

where ρ_f_ is the fluid density, ε_F_ is the local volume fraction of the fluid, **v**_F_ is the fluid velocity vector, and *t* is time. Similarly, the conservation of momentum is

4 $$ \frac{\partial }{\partial t}\left({\varepsilon}_F\cdot {\rho}_F\cdot {\mathbf{v}}_{\boldsymbol{F}}\right)+\nabla \left({\varepsilon}_F\cdot {\rho}_F\cdot {\mathbf{v}}_{\mathrm{F}}\cdot {\mathbf{v}}_{\mathrm{F}}\right)=-{\varepsilon}_F\cdot \nabla \mathrm{p}-\nabla \cdot \left({\varepsilon}_F\cdot {\boldsymbol{\uptau}}_F\right)+{\varepsilon}_F\cdot {\rho}_F\cdot \mathbf{g}-{\mathbf{S}}_M $$

**S**_**M**_ is the momentum transfer source term. The heat and mass transfer is identical to the momentum exchange. For detailed information about the coupling mechanism, see Jajcevic *et al.* (38).

The energy conservation equation for the gas phase is:

5 $$ \frac{\partial }{\partial t}\left({\varepsilon}_F\cdot {\rho}_F\cdot {c}_{p,F}\cdot {\mathrm{T}}_F\right)+\nabla \left({\varepsilon}_F\cdot {\rho}_F\cdot {\mathbf{v}}_{\mathrm{F}}\cdot {c}_{p,F}\cdot {\mathrm{T}}_F\right)=\nabla \cdot \left({\varepsilon}_F\cdot {k}_F^{eff}\cdot \nabla {\mathrm{T}}_F\right)+{\mathbf{S}}_E $$

where *c*_*p*, *F*_ is the gas heat capacity, T_*F*_is the gas temperature, *S*_*E*_is the inter-phase energy transfer, and ${k}_F^{eff}$is the effective thermal conductivity calculated as

6 $$ {k}_F^{eff}=\frac{1-\sqrt{1-{\varepsilon}_F}}{\varepsilon_F}\cdot {k}_F $$

The scalar transport of an arbitrary gaseous species “i” is described by the following equation:

7 $$ \frac{\partial }{\partial t}\left({\varepsilon}_F\cdot {\rho}_F\cdot {\boldsymbol{\mu}}_{\boldsymbol{i},\boldsymbol{vap}}\right)+\nabla \left({\varepsilon}_F\cdot {\rho}_F\cdot {\boldsymbol{\mu}}_{\boldsymbol{i},\boldsymbol{vap}}\right)=\nabla \cdot \left({D}_{eff}\nabla \cdot \left({\varepsilon}_F\cdot {\rho}_F\cdot {\boldsymbol{\mu}}_{\boldsymbol{i},\boldsymbol{vap}}\right)\right)+{\mathbf{S}}_{i, vap} $$

***μ***_***i,vap***_ is the mass fraction of the gas species, D_eff_ is the diffusion coefficient, and ***S***_***i,vap***_ is the gas species source term. Forgber *et al.* (39) have provided an in-depth overview of the models used and validated the momentum, heat, and mass transfer models for spheres.

### Bi-Convex Tablet Model

**Fig. 1:**
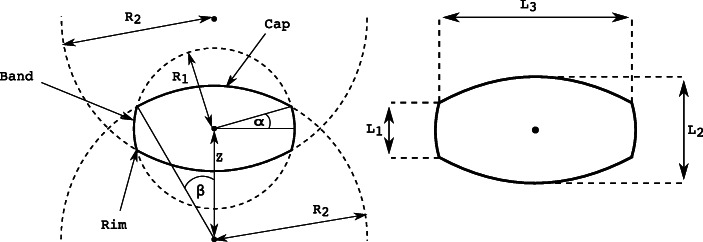
Construction of the biconvex tablet shape, including all symbols and expressions used. The image is adapted from Kureck *et al.* (40)

A bi-convex tablet model was developed to accurately account for the given tablet shape. The tablet has a diameter of 9.8 mm (L_3_) and a height of 4.3 mm (L_2_) with a rim thickness of 2.6 mm (L_1_). The tablet has a weight of 400 mg and a target hardness of 160 N. For CFD-DEM simulations, the tablet shape is modeled by three overlapping spheres and assumes that all tablets have the same size. The three spheres consist of one central smaller sphere describing the rim surface and two larger ones describing the cap. Figure 1 shows a sketch of the approximation *via* three overlapping circles.

Approximation *via* three overlapping spheres has advantages over other widely used shape approximations (*e.g.*, the multi-sphere model and polyhedral shape model). Since only three spheres are needed to approximate the shape (*vs.* eight spheres typically applied under the traditional multi-sphere approach (24,28)), the amount of memory required for the simulation is reduced as is the time for contact detection, decreasing the computational expense, while maintaining high accuracy of the simulation. Approximating the tablet shape without adding any artificial roughness as done *via* multi-sphere and polyhedral models enable fast simulations. For details of contact detection and force calculations, see Kureck *et al.* (40). Due to the non-spherical nature of the tablets, a specific drag model was developed for the coupling algorithms.

### Drag Force Model

In contrast to spherical particles, the calculation of the drag force acting on a tablet requires special consideration. There are many ways to compute the drag coefficient for spheres in coupled CFD-DEM simulations (15,41,42). Several models are available for calculating the drag force acting on non-spherical elements (43,44). Typically, these drag models only take into account a single particle in relatively dilute flows. However, for dense regimes of non-spherical particles, such as tablets in a coater, the correlation is not well established and is difficult to determine. Lattice Boltzmann methods are often used to calculate the fluid forces acting on the particles (45), *i.e.*, a model with varying numbers of fitting parameters is developed to correlate the drag on the particles. The problem with these approaches is that the fitting parameters have limited applicability to other shapes. Generally, several parameters have to be fitted to each particle shape. In this work, the drag coefficient calculation for non-spherical particles of Hölzer and Sommerfeld (44) for a single non-spherical particle is combined with the drag force model of Rong *et al.* (46,47). This model covers the single particle flow and the zones with a high packing fraction.

8 $$ {\overrightarrow{\boldsymbol{F}}}_i^d=\frac{1}{2}\cdot {C}_D\cdot {\rho}_F\cdot {A}_{\perp}\cdot {\varepsilon}_F^2\cdot \varphi \left({d}_i,{x}_i,{\varepsilon}_F\right)\cdot \left|{\overrightarrow{\boldsymbol{v}}}_F-{\overrightarrow{\boldsymbol{v}}}_{P,i}\right|\cdot \left({\overrightarrow{\boldsymbol{v}}}_F-{\overrightarrow{\boldsymbol{v}}}_{P,i}\right)\cdot {\varepsilon_F}^{-\left(\beta \left({\varepsilon}_F,\left\langle \mathit{\operatorname{Re}}\right\rangle \right)+\left(\lambda \left(\phi, \left\langle \mathit{\operatorname{Re}}\right\rangle \right)\right.\right.} $$

*C*_*D*_ is the drag coefficient, *A*_⊥_ is the projected area perpendicular to the airflow direction. *A*_⊥_ is calculated based on an approximation algorithm, which interpolates the projected area of the tablets based on the mean angle and surface area computed at the beginning of the simulation. *φ*(*d*_*i*_, *x*_*i*_, *ε*_*F*_) is a correction factor, the coefficient depends on the particle characteristic diameter (d_i_), particle type fraction *x*_*i*_ and void fraction *ε*_*F*_. Since only one type of particle is considered in this work, *φ*(*d*_*i*_, *x*_*i*_, *ε*_*F*_) is set to 1. For simulations with different particle shapes or sizes, this parameter has to be defined *via* calibration with highly resolved DNS-CFD simulations with varying packing fractions, void fractions, and diameters. The drag coefficient is calculated as:

9 $$ {C}_D=\frac{8}{\mathit{\operatorname{Re}}}\cdot \frac{1}{\sqrt{\phi_{\perp }}}+\frac{16}{\mathit{\operatorname{Re}}}\cdot \frac{1}{\sqrt{\phi }}+\frac{3}{\sqrt{\mathit{\operatorname{Re}}}}\cdot \frac{1}{\phi^{\frac{3}{4}}}+0.42\cdot {10}^{0.4{\left(-\log \left(\phi \right)\right)}^{0.2}}\frac{1}{\phi_{\perp }} $$

Re is the Reynolds number of the surface area equivalent sphere, *ϕ* is the sphericity of the tablet and *ϕ*_⊥_ is the crosswise sphericity. The sphericity is the ratio of the tablet volume to the sphere volume with the same surface area. The crosswise sphericity is calculated through *A*_⊥_ and is the exposed surface area ratio of the tablet volume to the surface area of a sphere with the same volume.

10 $$ \mathit{\operatorname{Re}}=\frac{v_{F,P}\cdot {d}_p}{\upsilon } $$

*v*_*F*, *P*_ is the relative velocity between the fluid and the tablet, *d*_*p*_ is the diameter of the area equivalent sphere and *υ* is the kinematic viscosity. *β* is a correction factor for a low-void fraction, taking into account the void fraction and Reynolds number,

11 $$ \beta \left({\varepsilon}_F,\mathit{\operatorname{Re}}\right)=2.65\cdot \left({\varepsilon}_F+1\right)-\left(5.3-3.5\cdot {\varepsilon}_F\right)\cdot {\varepsilon}_F2\cdot \exp \left[\frac{1}{2}\cdot \left(1.5\cdot \log \left(\mathit{\operatorname{Re}}\right)\right)2\right] $$

*λ* is a correction factor that considers the sphericity *ϕ*, as well as the Reynolds number,

12 $$ \lambda \left(\phi, \mathit{\operatorname{Re}}\right)=\left(1-\phi \right)\left\{C-D\cdot {e}^{-0.5\cdot {\left(3.5-\log \mathit{\operatorname{Re}}\right)}^2}\right\} $$

C is based on a linear regression of drag forces,

13 $$ C\left(\phi \right)=39\cdot \phi -20.6 $$

D is also based on the above linear regression,

14 $$ D\left(\phi \right)=101.8\cdot {\left(\phi -0.81\right)}^2+2.4 $$

In addition to the drag force, a CFD-DEM simulation of non-spherical particles has to consider the fluid lift force, C_L_. To calculate C_L_, the lift coefficient is computed. Correlations for the lift coefficient typically link the lift coefficient to the drag coefficient and the relative angle of fluid velocity fields to the particle orientation φ:

15 $$ {C}_L={\mathrm{C}}_{\mathrm{D}}\sin 2\left(\upvarphi \right)\cdot \cos \left(\upvarphi \right) $$

Torque resulting from the non-sphericity of the tablets should not be neglected. To calculate it, the center of gravity, x_cp_, has to be computed:

16 $$ {\mathrm{x}}_{\mathrm{cp}}=\mathrm{L}\left(3/4\right)\cdot \Big(\sin \left(\upvarphi \right)/\left(4+\uppi\ \cos \left(\upvarphi \right)\right) $$

The torque acting **(M**_***F***_**)** on the tablets due to the fluid-tablet interaction can be calculated by multiplying the center of gravity by the lift force ${\overrightarrow{\boldsymbol{F}}}_{Lift}$, the drag force ${\overrightarrow{\boldsymbol{F}}}_{Drag}$ **an**d other forces ${\overrightarrow{\boldsymbol{F}}}_{other}$

17 $$ {\mathbf{M}}_{\boldsymbol{F}}={\mathrm{x}}_{\mathrm{cp}}\left({\overrightarrow{\boldsymbol{F}}}_{Lift}+{\overrightarrow{\boldsymbol{F}}}_{Drag}+{\overrightarrow{\boldsymbol{F}}}_{other}\right) $$

### Heat and Mass Transfer

The heat and mass transfer is realized *via* source terms, *S*_*E*_ and *S*_*vap*_. In this work, it is assumed that the air is not saturated and no condensation occurs during the coating process.

The energy source term, *S*_*E*_, is calculated based on the heat transfer coefficient, tablet surface *A*_*P*_, and the difference between the tablet and film coating suspension temperatures (*T*_*P*_ − *T*_*F*_) as follows:

18 $$ {S}_E={h}_{FP}\cdot {A}_P\cdot \left({T}_P-{T}_F\right) $$

19 $$ {h}_{FP}=\frac{Nu_P\cdot {\lambda}_F}{d_P} $$

The heat transfer coefficient, *h*_*FP*_, is a function of the Nusselt number, *Nu*_*P*_, fluid heat conductivity, *λ*_*F*_, and the specific particle diameter, *d*_*P*_. For tablets, a specific diameter is the diameter of the area-equivalent sphere.

20 $$ {Nu}_P=\left(7-10{\varepsilon}_F+5{\varepsilon}_F^2\right)\left[1+0.7{\mathit{\operatorname{Re}}}^{0.2}{\mathit{\Pr}}^{0.33}\right]+\left(1.33-2.4{\varepsilon}_F+1.2{\varepsilon}_F^2\right){\mathit{\operatorname{Re}}}^{0.7}{\mathit{\Pr}}^{0.33} $$

The Nusselt number is a function of the void fraction Reynolds and Prandtl numbers. The Prandtl number is the ratio of the kinematic viscosity the thermal diffusivity *a*:

21 $$ \mathit{\Pr}=\frac{\upsilon }{a} $$

Mass transfer is modeled in analogy to the heat transfer:

22 $$ {\dot{m}}_P={k}_m\cdot {A}_{P,C}\cdot \left({w}_P-{w}_F\right) $$

Mass transfer ${\dot{m}}_P$ is calculated *via* the mass transfer coefficient, *k*_*m*_, the coated area, *A*_*P*, *C*_, and the difference between the equilibrium humidity at the tablet surface temperature, *w*_*P*_, and the surrounding gas, *w*_*F*_.The wet surface area is calculated according to the model proposed by Kariuki *et al.* (48).

23 $$ {k}_m=\frac{Sh_P\cdot {D}_{eff}}{L} $$

The mass transfer coefficient is a function of the Sherwood number, Sh, and the binary diffusion coefficient.

24 $$ {Sh}_P=\left(7-10{\varepsilon}_F+5{\varepsilon}_F^2\right)\left[1+0.7\cdot {\mathit{\operatorname{Re}}}^{0.2}\cdot {Sc}^{0.33}\right]+\left(1.33-2.4{\varepsilon}_F+1.2{\varepsilon}_F^2\right)\cdot {\mathit{\operatorname{Re}}}^{0.2}\cdot {Sc}^{0.33} $$

The Sherwood number (Sh_P_) is a function of the void fraction and the Reynolds (Re) and Schmidt (Sc) numbers (49). The Schmidt number is the ratio of the kinematic viscosity and the diffusion coefficient.

25 $$ Sc=\frac{\upsilon }{D_{eff}} $$

The presented evaporation model takes the surface evaporation (first phase evaporation) on the tablet surface into account. The influence of spray drying, liquid absorption of the tablet core, second, and third stage drying are neglected in the presented model.. It is also assumed that the tablet properties do not change during and after spraying of the coating liquid, except for mass.

### Spray Coating Model

To model the spray, an inline ray tracing algorithm similar to the approach proposed by Toschkoff *et al.* (26,50) was used to detect the coated tablets. This algorithm requires a point of origin, opening angles, droplet size distribution, spray interval, and direction vector as input variables. For each spray nozzle, a point of origin for each spray nozzle and a direction of the spray vector has been defined. The number of rays is a function of droplet size and spray interval time. This approach neglects the momentum, heat and mass transfer from the spray to the surrounding fluid. All mass and energy are only transferred between the spray and the tablet or between the tablet and the fluid. From the point of origin in the direction of the spray, tablets in the way of the spray vector are monitored. If a ray detects a tablet, the spray mass is given by the droplet radius, the spray composition, and the density is transferred to the tablet. The spread is calculated *via* the spread function during the simulation run. The coated surface area is calculated using the above-mentioned algorithm of Kariuki *et al.* (48), which is also applied to calculate the evaporation rate. For more information about this approach, please refer to Forgber *et al.* (39)**.**

## ConsiGma® Coater

In the following subsections, the ConsiGma® coater is introduced in detail, from the coating geometry to an explanation of the process and experimental data.

### Coater Geometry

The coating process in the ConsiGma® coater involves the fast coating of small batches with a consistent coating quality in series. This approach has several advantages over truly continuous and traditional processes. Experiments for setting up a new product or optimizing an existing one are fast and do not require a lot of material. Process settings are tested in small sub-batches, and a continuous process is achieved by recreating the optimal process conditions over and over. Additionally, no start-up (*e.g.*, pre-heating and cooling) or end effects, typically associated with continuous processing, are present and the equipment’s footprint is smaller.

**Fig. 2:**
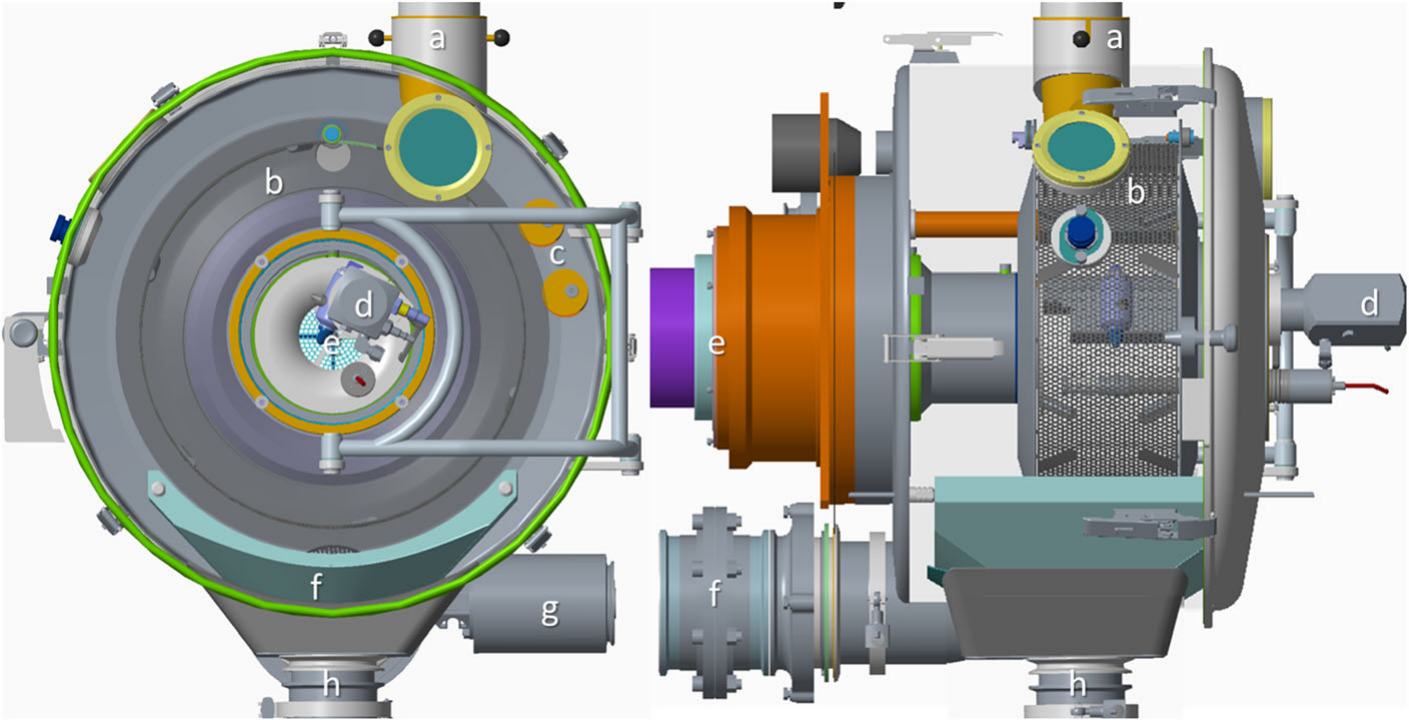
ConsiGma® coater, including the supporting surroundings: (**a**) inlet funnel; (**b**) drum; **c** air knives; (**d**) spray nozzle; (**e**) air inlet; (**f**) air outlet; (**g**) temperature/ humidity sensor; (**h**) outlet funnel

An overview of the most important ConsiGma® tablet coater parts is provided in Fig. 2. The coating drum (b) is placed in a housing and loaded over a movable lid and chute (a). The adjustable spray nozzle is centrally mounted and is typically in a 12 o’clock position (d). It can be moved vertically and horizontally and rotated around its center. Air is introduced into the drum coater through a central pipe behind the coating wheel (e). Conditioned inlet air knives, which are installed outside of the drum coater between 2 and 3 o’clock (c), dislodge the tablets from the wall and guide them to the spray zone and provide additional drying air. When the coating process is finished, the tablets are discharged from the bottom of the drum (f, h). In addition, the sensors (g) measure the temperature and humidity of air exiting from the outlet air pipe (f) behind the drum coater.

The CFD-DEM simulations only consider the drum interior (b). The drying air inlet was placed on the side of the drum, and the air knives were modeled as mass inlets on the drum surface. The remaining drum surface was set as the outlet region. The spray nozzle arm and the accompanying light source were included in the simulation volume. In the simulation, the surrounding housing and equipment were neglected, as well as the heat loss to the surrounding air.

**Fig. 3:**
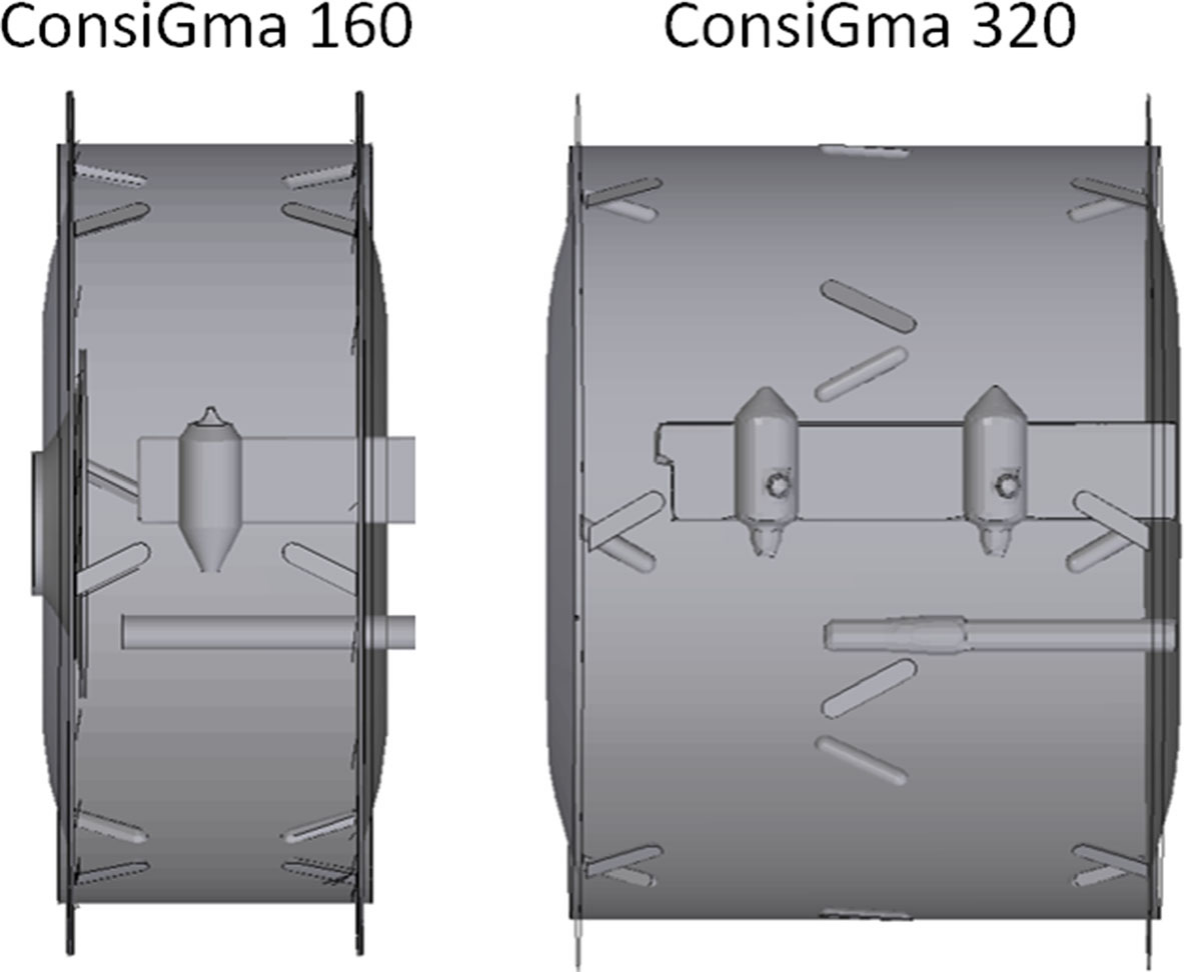
Coater geometry of the ConsiGma 160 (left) and 320 (right) coater

The ConsiGma® tablet coater drum has a diameter of 44.5 cm and a depth of either 160 mm or 320 mm (Fig. 3) determined by the drum insert, hereafter denoted as ConsiGma® 160 and ConsiGma® 320 respectively. The drums can be loaded with 2.5 to 7 kg of tablets depending on the tablet shape and the drum used. The 160 mm drum employs one spray nozzle (Fig. 3), while the 320 mm coater uses two. The spray nozzle(s) are set up so that the spray zone covers nearly the entire drum depth. This minimizes the need for fast axial tablet mixing, and the tablet bed has to be mixed only in a radial direction. ConsiGma® 320 has central gripper bars in addition to the gripper bars on the side of the drum to stabilize the tablet bed and accelerate the ring formation.

### Process Description

**Fig. 4:**
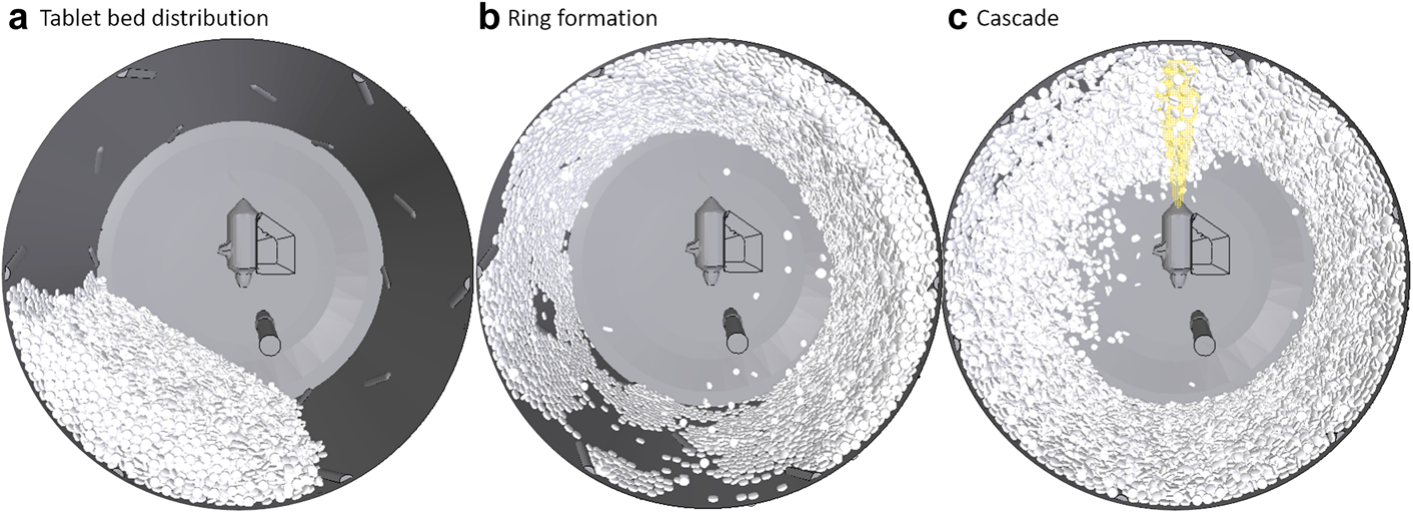
Stages of the coating process, loading/tablet bed distribution (**a**), ring formation (**b**), and tablet cascade with spray (**c**)

The coating process in the ConsiGma® coater can be divided into seven stages: loading, bed distribution, ring formation, cascade formation, spray coating, drying and discharging. The ConsiGma® coater is loaded *via* a hopper above the coater. When the loading stage is completed, the drum lid is closed and the coater is rotated at a low speed (*e.g.*, 5 rpm) to evenly distribute the tablet bed (Fig. 4a). After this, the drum accelerates to up to 115 rpm. High rotation rates enable the tablets to form a ring along the drum wall due to the centrifugal force that exceeds the gravitational force (Fig. 4b). When the tablet bed forms a steady ring, the rotation rate is reduced to 88–95 rpm depending on the tablet shape, the material properties, the drum load, and the drum type. The air knives inject air from the side and the tablet cascade is formed in preparation for spraying. Once a stable cascade is formed, the spray nozzle(s) are turned on to apply the coating solution (Fig. 4c) onto the tablets until the target quantity of suspension and the associated mass gain is reached. Finally, the tablets dry for a short time before the drum rotation stops and the tablet bed is allowed to collapse. After that, the lid at the bottom of the drum coater is opened and the coated tablet bed is discharged. The remaining tablets are emptied by slowly rotating the drum. A typical coating cycle lasts about 10 min depending on the target mass gain and the spray rate.

**Fig. 5:**
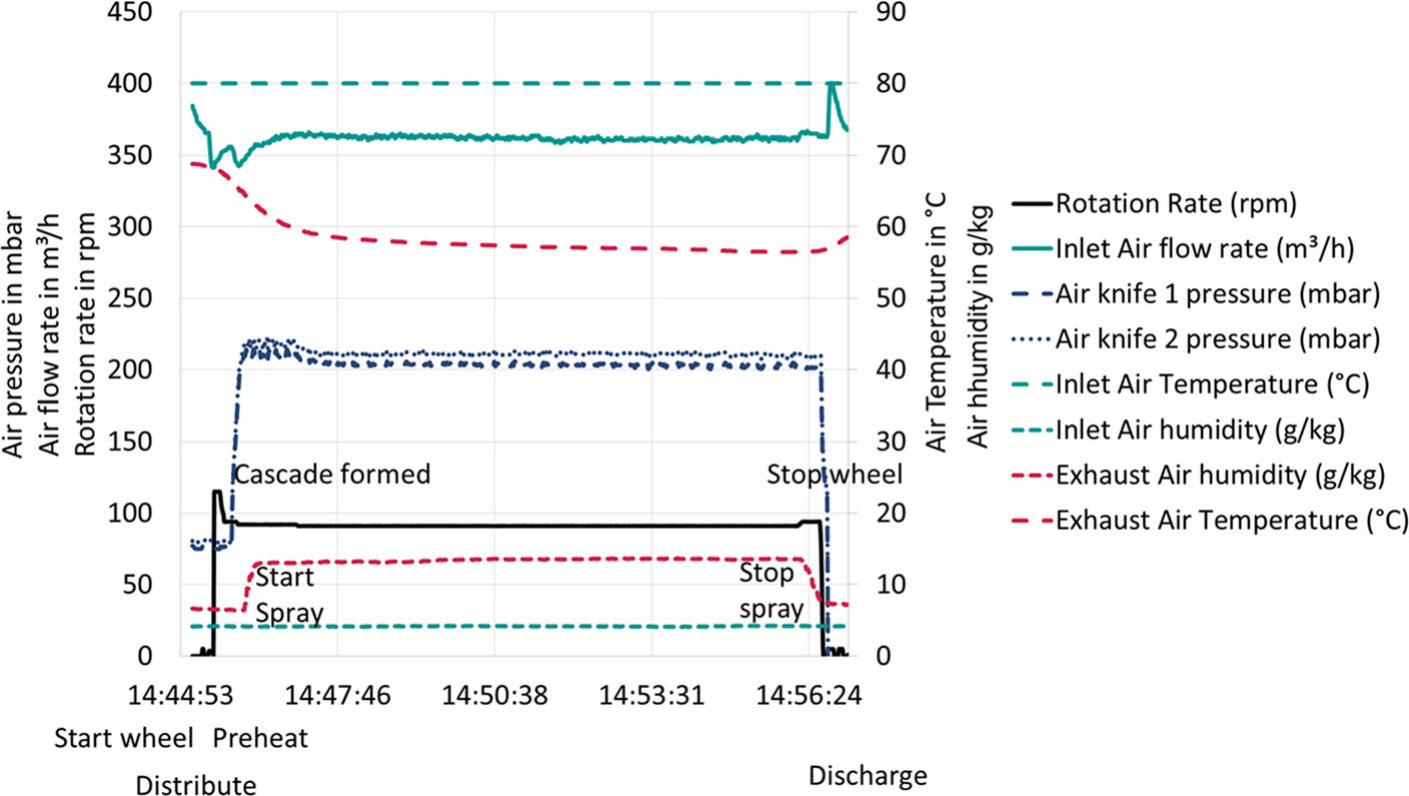
Typical coating process in the ConsiGma® coater

Figure 5 shows typical process data as read out from the tablet coater, including rotation rate, inlet air flow rate, air knife pressures, inlet temperature and humidity, and outlet air temperature and humidity. In the first 30 s, the tablets are loaded into the drum, which slowly rotates to distribute the tablet bed. After that, the rotation rate is increased to 115 rpm. In this phase, the tablets are distributed along the drum wall and form a ring. After 20 s, the rotation rate is reduced to 93 rpm and, shortly after, the air knives are started and the air knife pressure increases from its background pressure of 80 mbar to 220 mbar, which is kept constant during the coating process. In this example, the rotation rate is reduced in two steps from 93 rpm to 92 rpm and 91 rpm. This is needed due to changing tablet – drum friction, *i.e.*, the friction between the tablet and the drum changes as a function of the applied coating. The outlet air temperature decreases from 70°C to 60°C and the air humidity increases from 6 g/kg to 14 g/kg of air. After 11 min, the coating process is completed and the tablets are discharged from the coater wheel. After that, the outlet air temperature increases and the humidity decreases.

### Experimental Setup and Operating Points

During the experimental runs, four parameters are tracked to validate the simulation model. First, the forces acting on the tablets are recorded to validate the tablet bed dynamics. Second, the coating mass variability is assessed by tracking the mass gain of 30 tablets across the coating process. Third, the influence of spray rate, drying air flow rate and temperature on the outlet air humidity is analyzed. Fourth, the influence of spray rate, drying air flow rate and temperature on the outlet air temperature is established.

**Fig. 6:**
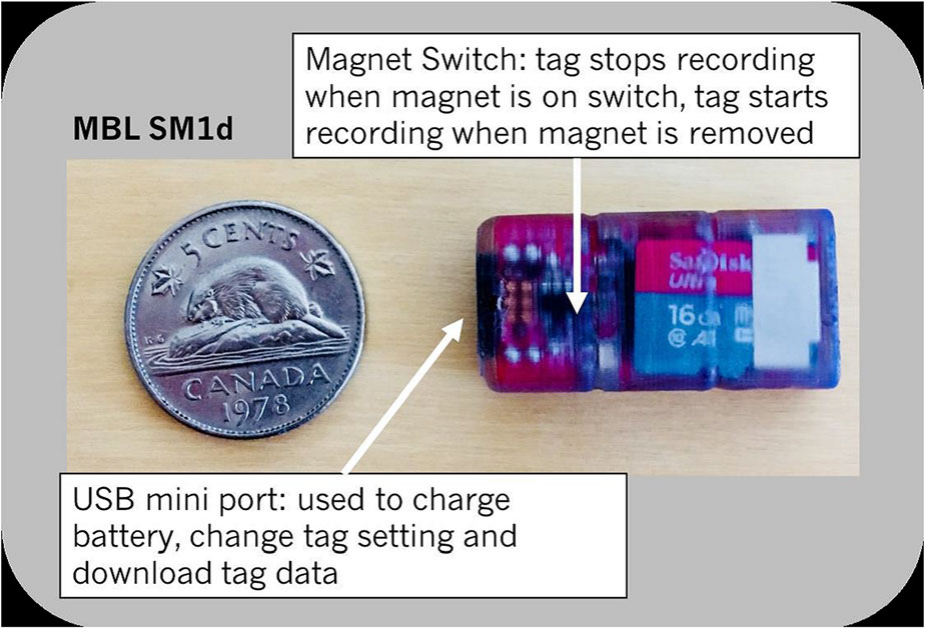
Size comparison of the Maritime BioLogger with a 5 cent coin (image from the manual)

**Fig. 7:**
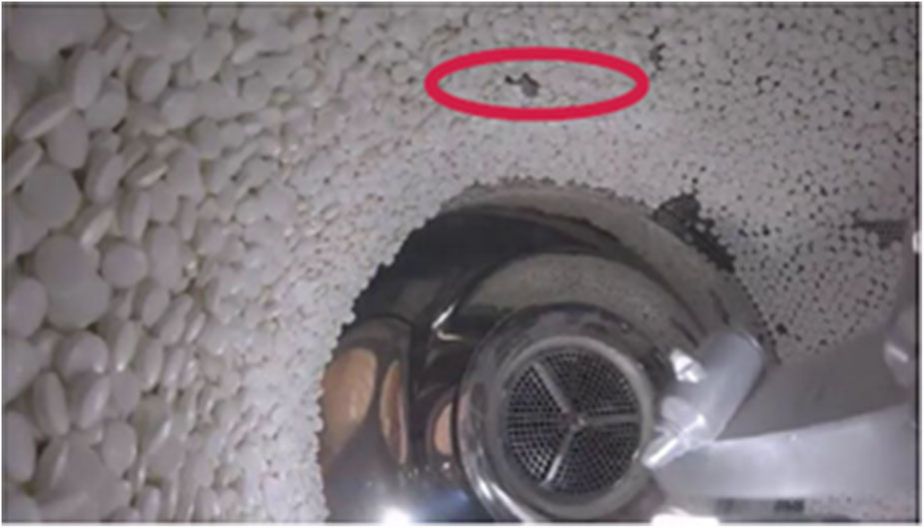
Maritime BioLogger inside the tablet bed in the ConsiGma® 320 tablet coater

To validate the tablet bed dynamics of the simulation model, the acceleration of model tablets is compared to the experimental results. To monitor the forces inside the ConsiGma® coater experimentally, an accelerometer from Maritime BioLogger (https://maritimebiologgers.com/) (Fig. 6) is placed in the system to track the acceleration in three dimensions every 0.02 s. Due to the small size, it is assumed that the forces that the accelerometer experiences are similar enough to the accelerations experienced by the tablets during the coating process (Fig. 7). The data are stored on a MicroSD and evaluated after the experiment is completed.

The coating mass variability is tracked by marking and weighing 30 individual tablets before starting the coating process. The tablets are coated for 1%, 2%, 3%, and 4% coating mass gain. After the coating process is finished, the marked tablets are weighed again and the coefficient of inter-tablet coating variation is calculated.

The outlet temperature and humidity are recorded automatically during the coating process inside the ConsiGma® coater. Both values are saved every 2 s and can be accessed either during the coating process or in subsequent analysis. The tablets are dried for up to 30 s and then discharged. The tablet temperature is measured after the coating process is finished using an infra-red camera and a distance thermometer.

Altogether, six experiments were performed to validate the simulation results for the heat and mass transfer. In the experiments, the drum load, rotation rate, and air knife pressure were kept constant, while the drying air flow rate, temperature, spray rate, and the coater size are varied (Fig. 3). The simulations were run until the tablet, air humidity and temperature reached a steady state. The DoE is provided in Table I.

**Table I:** Process Parameters of the Experiments and Simulations Performed

Process property (DoE number)	Coater type	Load (kg)	Rotation rate (rpm)	Spray rate (g/min)	Number of nozzles	Drying air flow rate (m^3^/h)	Drying air temperature (°C)
1	160	3	90	75	1	180	60
2	160	3	90	60	1	180	80
3	160	3	90	45	1	210	90
4	320	6	93	75	2	300	60
5	320	6	93	60	2	360	80
6	320	6	93	45	2	420	90

## RESULTS

The primary goal of this work was to validate the CFD-DEM model of the coating process in the ConsiGma® tablet coater. The model was validated concerning the four most important parameters that define the tablet coating process. First, the tablet bed dynamics were analyzed. Changes in acceleration during the various phases were tracked using an accelerometer and compared to the simulation results. Second, the coating mass gain and distribution were assessed. Experiments were conducted by tracking the coating mass of 30 tablets for 1–4% coating mass gain. The mean coating mass and standard deviation were measured and used to calculate the coefficient of variation. The data were then compared to the simulation results. Third, the outlet air humidity was tracked. Six experiments were performed and recreated *in silico*. Process conditions were varied from wet to dry in two coater drum sizes. Additionally, the LoD of the tablets was tracked experimentally and compared to the simulation. Fourth, the outlet air temperatures were analyzed. Also, the tablet temperature during the coating process was evaluated in the simulation (without being compared to the experimental results).

### Forces

**Fig. 8:**
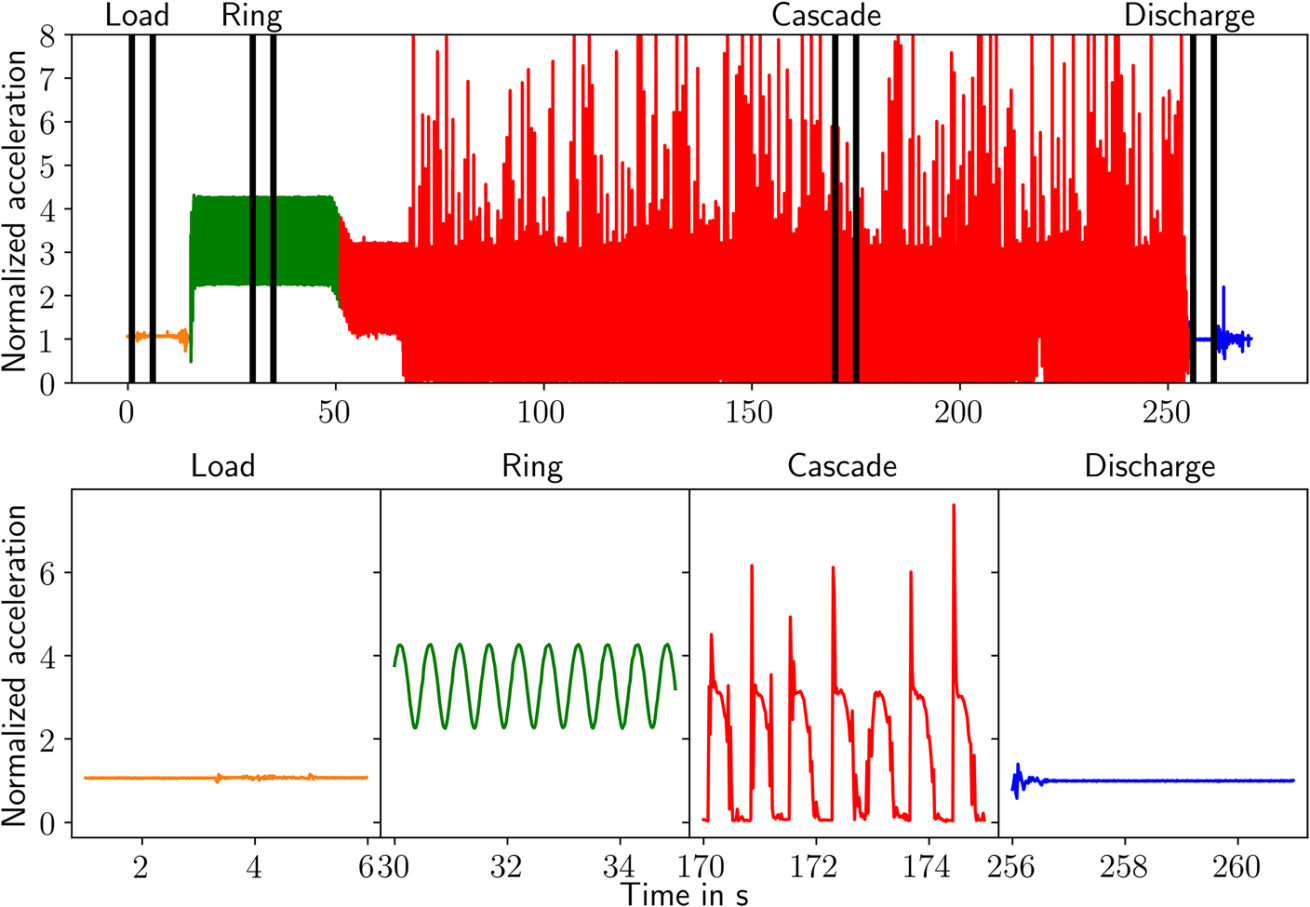
Accelerations recorded during an experimental run. The various phases of the coating process are shown in the subplots below: drum loading and tablet bed distribution, ring formation, cascade formation, and drum discharge

An accelerometer tracks changes in the acceleration during the various phases of the coating process. The results are shown in Fig. 8, which illustrates the following four stages: loading, ring formation, cascade formation, and discharge process. In the bottom row, 5 s of the four phases of the coating cycle in the ConsiGma® coater are shown in detail. The top row depicts the entire 260 s of the experimental run, including the transition from one phase to the next. The positions in time of the five seconds of interest are indicated by two black vertical lines. In this work, the acceleration is normalized using the gravity constant of 9.81 m/s^2^. During the load and tablet bed distribution phase, the accelerometer experiences accelerations of around 1 g, *i.e.*, mainly the gravitational pull. All other changes are minimal.

During the ring formation, the drum coater rotation rate reaches 115 rpm and a regular sine wave-like behavior can be observed. During this phase, the tablet bed is in a steady-state and the movement of the tablets relative to each other is almost zero. The various accelerations tracked correspond to the position of the accelerometer. High acceleration values indicate the bottom of the drum coater, with the centrifugal and gravitational forces working in the same direction. The low values correspond to the top of the drum coater, with the centrifugal force counteracted by the gravitational force. A full rotation in the ring phase takes around 0.5 s.

After the ring formation, the cascade phase begins. First, the rotation rate of the drum is reduced to 93 rpm. After 15 s, air knives are activated to push the tablets away from the drum wall and allow for a wider cascade as well as enhanced mixing in the reentry zone. During this phase, peaks of up to 8 g are visible as well as periods of almost zero acceleration changes. Periods of high acceleration are due to rapid changes in the acceleration indicating the reentry or contact of the accelerometer with the tablet bed and drum wall after the free fall of the cascade. The periods of no acceleration relate to the free fall inside the cascade. During the free fall, the gravity and the other accelerations seem to be in equilibrium. The accelerometer falls with the tablet bed cascade through approximately 1/3 of the drum coater with no change in the direction and velocity. A full rotation during the cascade phase takes around 0.7 s. After the cascade, the tablet bed collapses and the drum rotation is reduced to 5 rpm. Again, mainly the gravitational force is acting on the accelerometer.

**Fig. 9:**
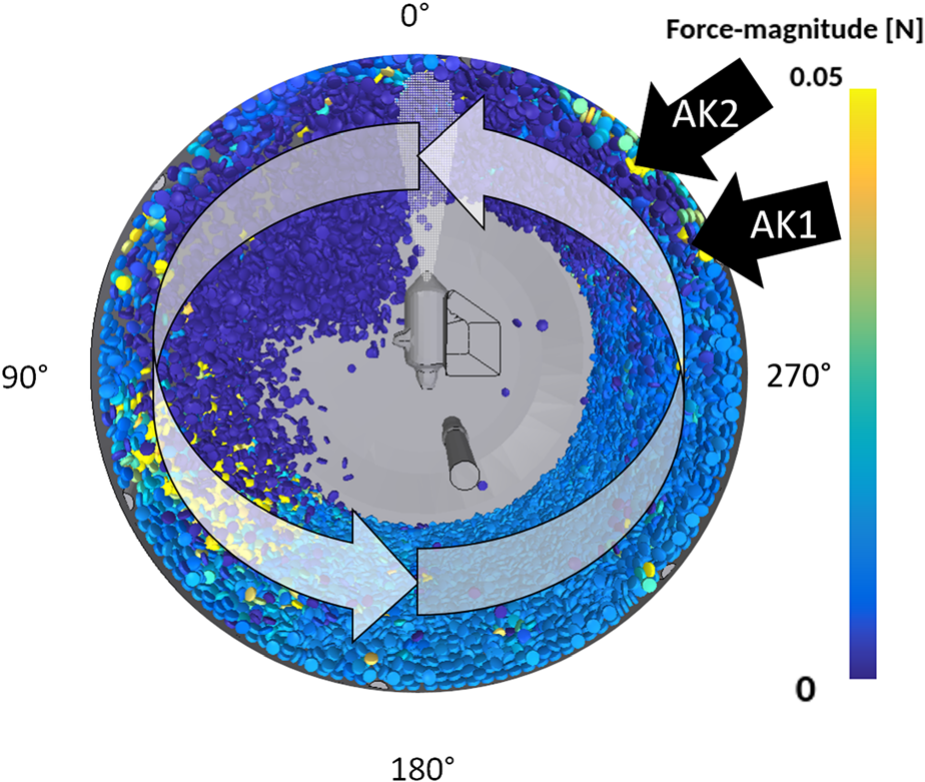
Visualization of the drum angle and the tablet forces

Figure 9 shows an image of the simulated tablet bed during the cascade phase. The tablets are colored according to the forces acting on them, and the angle degree of the coater positions is shown around the drum. 0° is at the top of the drum and the angle is rotated counterclockwise, just as the tablets during the coating process do. The tablets experience low/no forces during the cascade phase, high forces during the re-entry into the tablet bed and medium forces at the bottom of the drum.

**Fig. 10:**
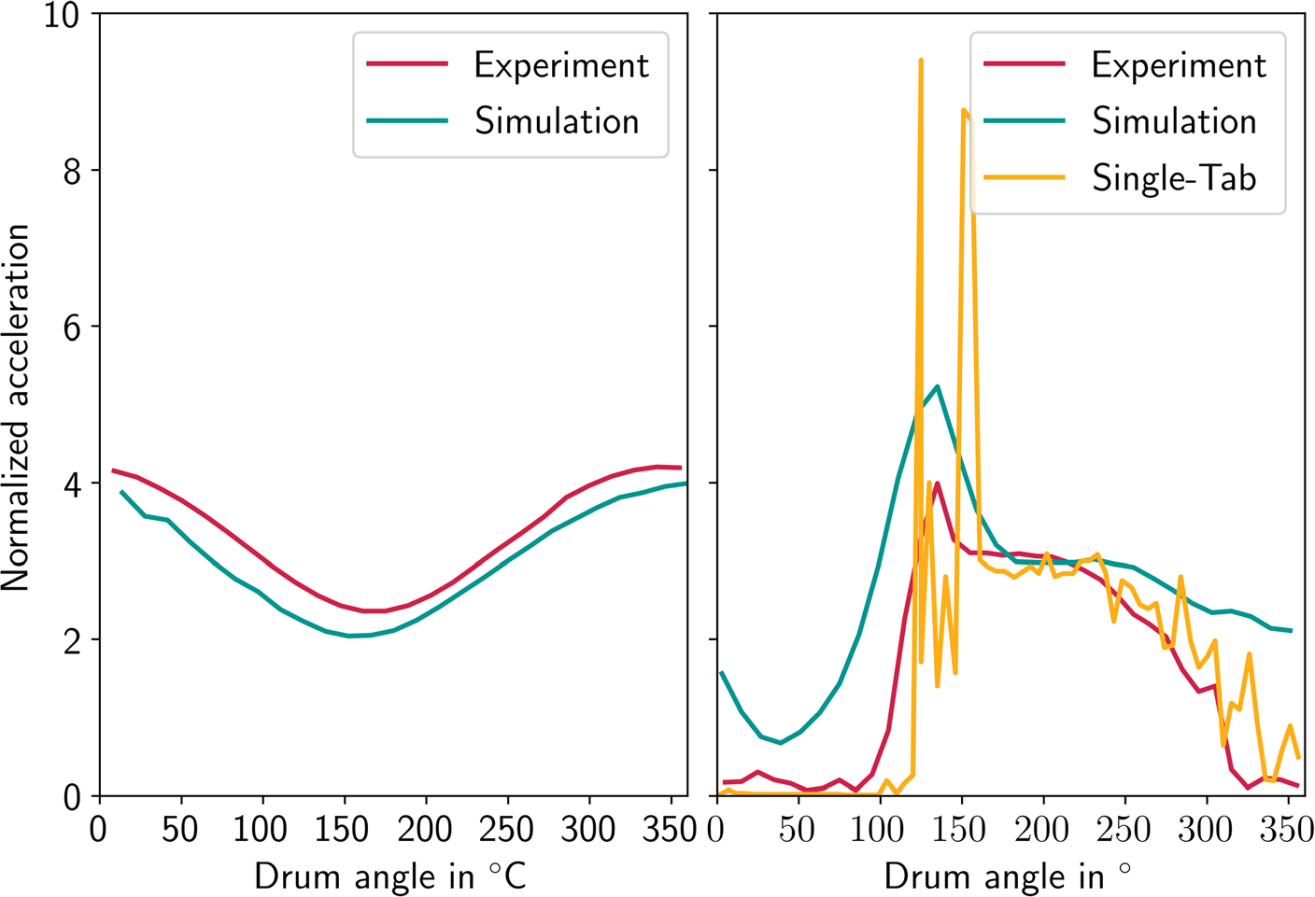
Experimental time-averaged forces (experiment) and comparison to time- and tablet-averaged forces acting on the tablets (simulation). In addition, time-averaged forces for a single tablet are shown based on the simulation data

Figure 10 shows the time-averaged acceleration values in the ring and cascade phases and a comparison between the simulation and experimental results. The simulation results are time- and spatially averaged over all tablets. On the left-hand side, the acceleration during the ring phase is shown. The experimental and simulation results agree well, as can be seen. An increase and a decrease in the acceleration from the bottom to the top of the drum and back are visible, as well as the magnitude of the acceleration changes.

On the right-hand side, the measured and computed accelerations during the cascade phase are shown, as well as the time-averaged acceleration changes of a single tablet during the simulation. The simulated and experimental results also agree well concerning the overall shape of the curves. Once again, the value at the bottom of the drum (180°) is in good agreement. In the simulation, the zone of reentry into the tablet bed between 90° and 150° is wider and the maximal forces are higher. The zone of free fall/low acceleration is wider in the experimental results than it is according to the time- and spatially averaged simulated results. In the simulations, some tablets adhered to the drum wall even during the cascade/free-fall period. Although this effect was also observed in the experiments for some tablets, it was not recorded by the accelerometer. A possible explanation is that due to its different size, shape, and mass the accelerometer segregates in the inner layer of the tablet bed. The inner layer of the tablet bed always detaches during the free-fall phase. Therefore tablets that stick to the drum wall have higher acceleration forces acting on them since the centrifugal forces from the drum rotation are still contributing. Thus, the mean force in this period is higher in the simulation. Since the shape and size of the accelerometer are different from those of the tablets, it tends to segregate and stay on top of the tablet bed. Therefore, it is more likely to detach from the drum wall and be engaged in the free-fall phase, while not all tablets follow that pattern.

Regardless of these factors, the experimental and simulated results agree well and confirm the accuracy of the simulation results, especially when the time-averaged data of a single tablet is compared to the time-averaged experimental results. For a single tablet, the experimental and simulated accelerations are in good agreement even in the free-fall zone, from 330° to 135°. The experimental, as well as the single tablet values, are time-averaged over ten rotations. Both the detached state during the free fall and the high acceleration in the reentry zones are captured in the simulation.

### Coefficient of Inter-Tablet Coating Variability

**Fig. 11:**
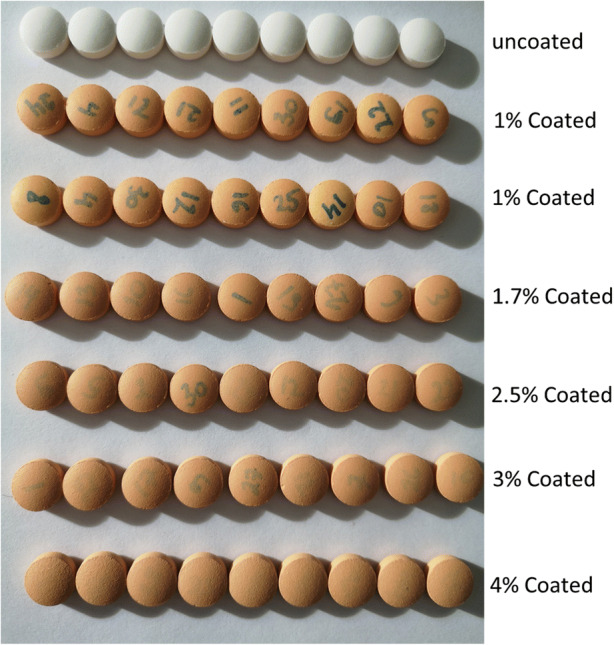
Uncoated and coated tablets with various mass gains

In the experiments, 30 tablets were weighed and numbered, and the starting and final coating mass gain were compared in the ConsiGma® 320, a drum load of 6 kg, drying airflow rate of 360 m^3^/h inlet air temperature of 80°C and a spray rate of 120 g/min. Figure 11 shows tablets coated with a mass gain of 1%, 1.7%, 2.5%, 3% and 4%. The mass gain of the marked tablets was used to calculate the mean coating mass and the standard deviation of the coating mass over time. In the case of 4% mass gain, the coating layer was too thick to identify all tablets, making it impossible to find all marked tablets.

**Fig. 12:**
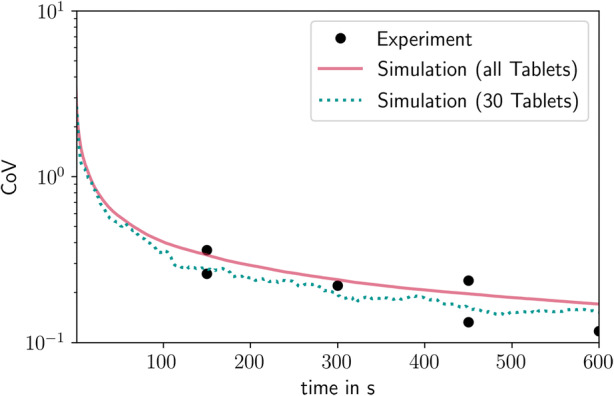
Evolution of the coefficient of variation and comparison between the experiment and the simulation. Both simulated and experimental results were run on the ConsiGma® 320 with a load of 6 kg drying air flow rate, 360 m^3^/h an inlet temperature of 80°C and a spray rate of 120 g/min

Changes in the CoV over the processing time are shown in Fig. 12. Six experiments with a process duration of 2.5, 5, 7.5, and 10 min were performed. This time corresponds to a coating mass gain of 1–4%. They are shown as black dots in the plot. The simulation results are represented by a solid red line for all tablets and a dotted blue line for 30 randomly selected tablets that were chosen to illustrate the influence of the number of tablets on the CoV variability. After an initial phase of 10 s, the simulation results show a steady decline over time with an exponent of −0.5, indicating that the coating process in the ConsiGma® matches the random tablet movement for a conventional batch tablet coating process (51). The simulation results agree well with the experimental values for the different evaluated coating periods.

**Fig. 13:**
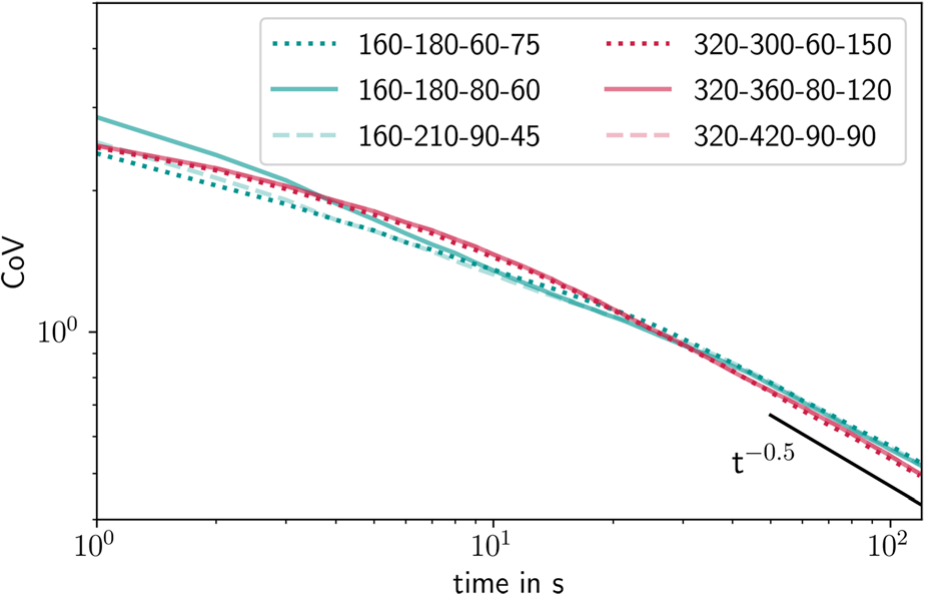
CoV in all simulated cases over time. The numbers in the legend refer to the coater wheel type, drying air flow rate (m^3^/h), drying air temperature (°C), and spray rate (g/min)

Figure 13 shows the simulated CoV for the six cases from Table I. The legend refers to the coater size in mm, drying airflow rate in m^3^/h, drying air temperature in °C, and spray rate in g/min. The results for the CoV follow a very similar trend. All simulations were run for a coating period of at least 120 s, with the CoV decrease reaching a steady state after about 20–30 s, or 30–45 coating rotations. Additionally, the plot shows that the ConsiGma® 160 and 320 tablet coaters behave similarly in terms of coating mass variability.

### Mass Transfer and Drying

**Fig. 14:**
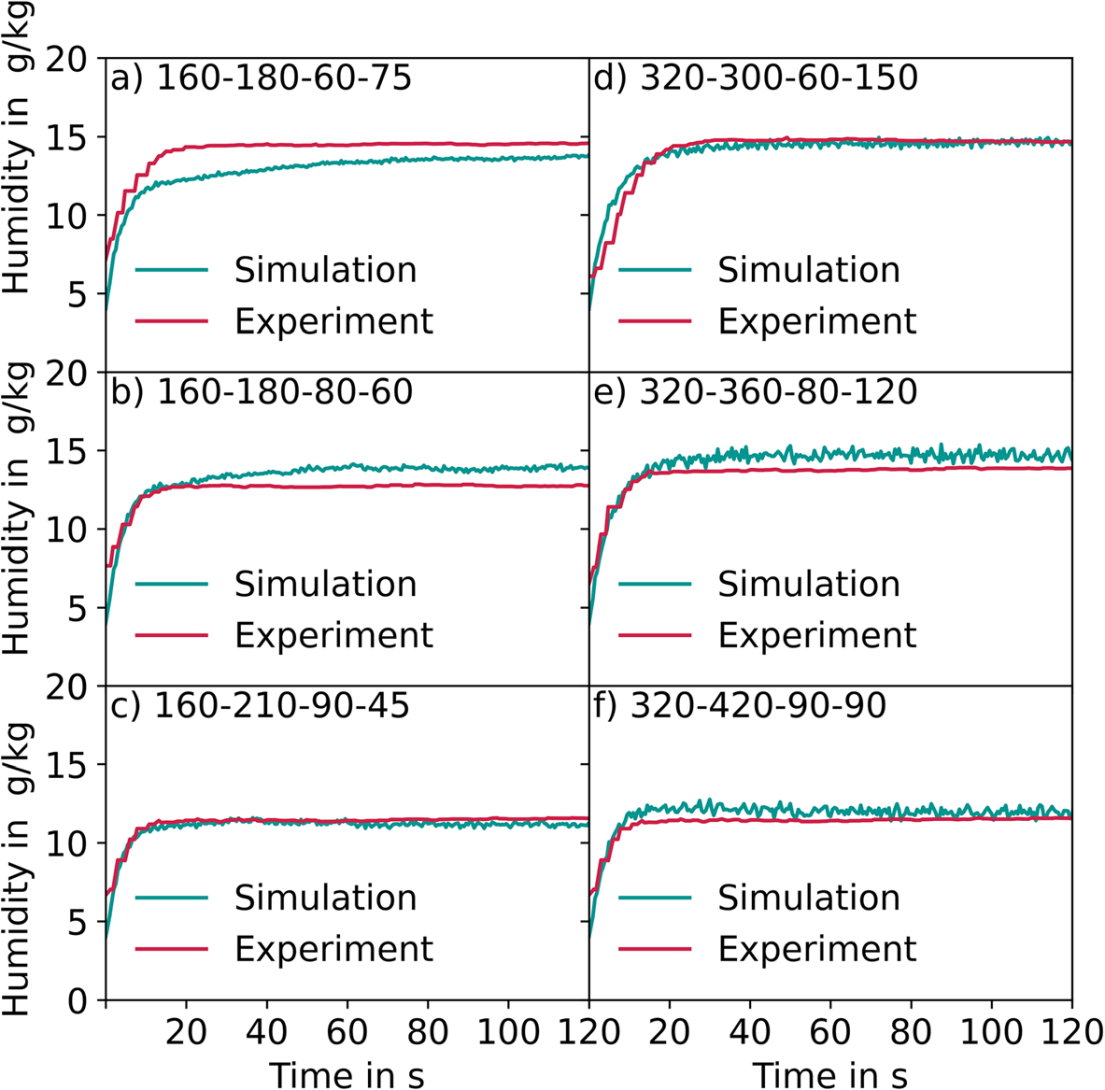
Comparison of the outlet humidity between the experiment and the simulation. (**a**) ConsiGma 160, drying air flow rate of 180 m³/h, temperature of 60 °C and spray rate 75 g/min, (**b**) ConsiGma 160, drying air flow rate of 180 m³/h, temperature of 80 °C and spray rate 60 g/min (**c**) ConsiGma 160, drying air flow rate of 210 m³/h, temperature of 90 °C and spray rate 45 g/min (**d**) ConsiGma 320, drying air flow rate of 300 m³/h, temperature of 60 °C and spray rate 150 g/min (**e**) ConsiGma 320, drying air flow rate of 360 m³/h, temperature of 80 °C and spray rate 120 g/min (**f**) ConsiGma 320, drying air flow rate of 420 m³/h, temperature of 90 °C and spray rate 90 g/min

Figure 14 shows the outlet air humidity over time after the spray nozzle is activated. The left and right columns show the values for ConsiGma® 160 and ConsiGma® 320, respectively, going from wet to dry cases. In the experimental system, the humidity is measured at the outlet behind the tablet coater. In the simulation, the outlet air humidity is measured at the drum surface The outlet air humidity increases for 10 s in the experiment in the simulation, from initial values of 4 g/kg to steady-state values of 15 g/kg in the wet cases and 11 g/kg in the dry cases. In the experiments, the values fluctuate between 4 and 6 g/kg. In the simulation, the inlet air humidity value in the simulation is set to 4 g/kg. Steady-state values match well in all cases, except for the wet case for ConsiGma® 160 (180 m^3^/h, 60°C, 75 g/min) in which the simulation underpredicts the outlet air humidity. In this case, the experimental inlet air humidity was higher than in the simulation, which explains the resulting higher outlet air humidity.

The results indicate that the drying air flow rate and temperature together with the spray rate influence the outlet humidity and the evaporation rate. Moreover, it can be concluded that the evaporation efficiency decreases with the decreasing temperature and airflow rate. The effect of inlet air humidity was not taken into account as the inlet air is dehumidified in all cases.

**Fig. 15:**
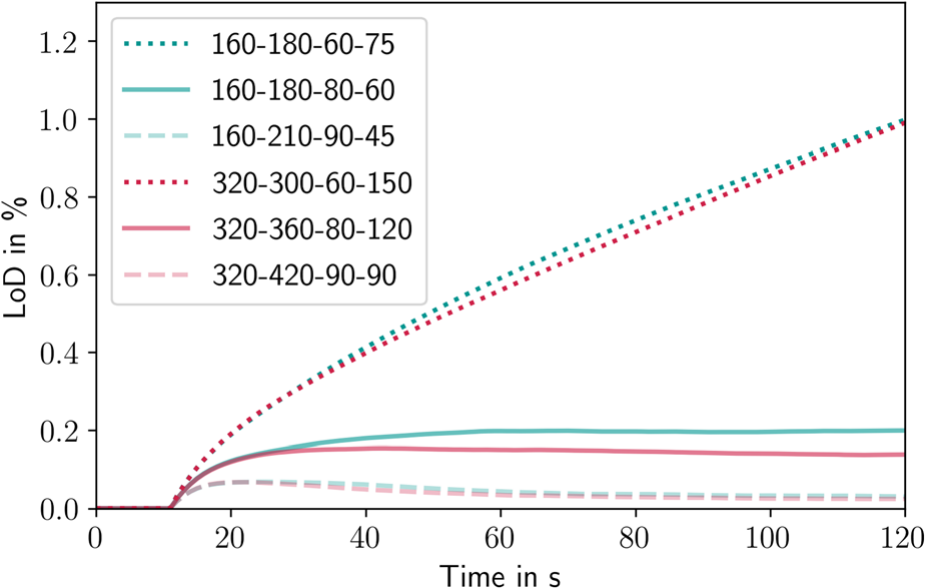
Mean liquid mass during the coating process inside the ConsiGma® coaters. The numbers in the plots refer to the coater wheel type, drying air flow rate (m^3^/h), drying air temperature (°C), and spray rate (g/min)

The mean LoD of the tablets in the simulation in all six cases investigated is shown in Fig. 15. LoD, in this case, means the amount of water that is not evaporated from the tablet surface in the simulation. There is no absorption model included in the evaporation model. In the simulations, the tablets are initialized without any liquid. When spraying begins after 12 s of process time, the liquid mass on the tablets increases in all cases. After 10 s of spray coating, the mean liquid mass of the tablets reaches a steady-state for dry and medium-dry conditions (160-180-80-60, 160-210-90-45, 320-360-80-120 and 320-420-90-90) and increases steadily for wet conditions (160-180-75-75 and 320-300-60-160). The steady-state mean LoD values in the dry case are 0.05%, 0.2% in the 160-180-80-60 case and 0.15% in the 320-360-80-120 case. Although neither of the wet cases (160-180-75-75 and 320-300-60-160) achieves a steady-state, the increase seems to get shallower over time and reaches an LoD of 1%. This means that in the wet case, the water on the tablets accumulates and does not evaporate fast enough. Such conditions may lead to tablet sticking and picking.

**Fig. 16:**
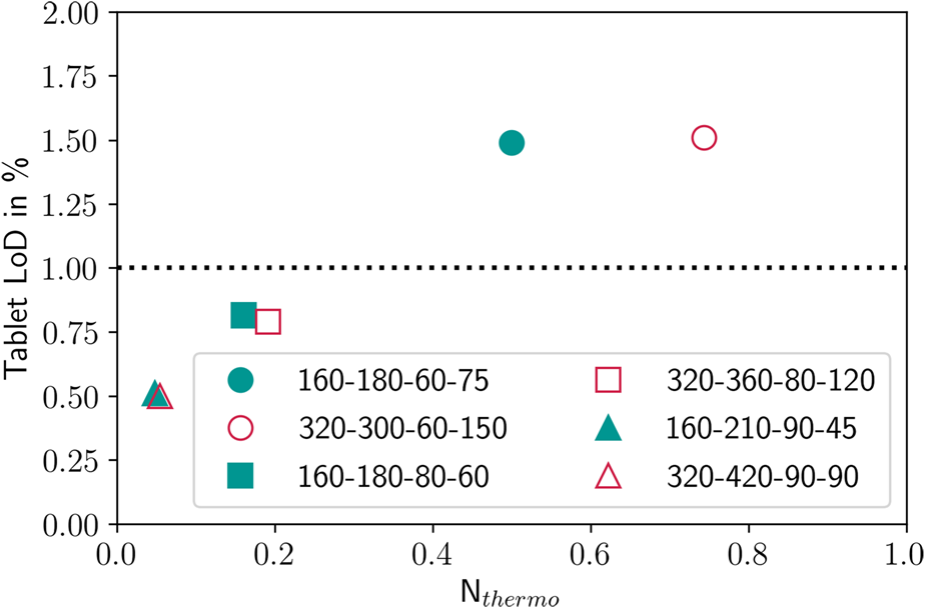
LoD of the tablets before and after the coating process. The numbers in the plots refer to the coater wheel type, drying air flow rate (m^3^/h), drying air temperature (°C), and spray rate (g/min)

An increase in the LoD under wet coating conditions was also observed in the experiments. Figure 16 shows the LoD of the tablets before and after coating. The uncoated tablets have an LoD of 1% due to the previous process steps and the surrounding air. The initial tablet LoD is plotted as a dotted line. Silverman *et al.* defined a scaling parameter for the thermodynamics of a film coating process, *N*_thermo_, as (52):

26 $$ {N}_{\mathrm{Thermo}}=\frac{{\dot{m}}_{\mathrm{water}}}{{\dot{V}}_{\mathrm{air}}\cdot {\rho}_{\mathrm{air}}\cdot \left({H}_{\mathrm{sat},\mathrm{bed},T}-{H}_{\mathrm{inlet},\mathrm{dew},\mathrm{point},T}\right)} $$

where ${\dot{m}}_{\mathrm{water}}$ is spray liquid mass flow rate [kg/h], ${\dot{V}}_{air}$is the drying air inlet volume flow rate [m^3^/h], *ρ*_*air*_ is the air density [kg/m^3^], *H*_sat, bed, *T*_ is the specific humidity of the inlet air at tablet bed temperature [kg/kg] and *H*_inlet, dew, point, *T*_ is the specific humidity of the inlet air at the inlet air dew point temperature [kg/kg]. Except for very wet coating conditions, the tablets leave ConsiGma® coater drier than when they entered it. Under very dry conditions, this means that the tablet LoD decreases from 1% to 0.5% and in the moderate cases to 0.8%. The tablets that leave ConsiGma® 320 are drier than those that leave ConsiGma® 160 at similar *N*_thermo_. Under wet conditions, the tablets retain some of the coating suspension. The LoD increases to 1.5% in both cases, although in the wet cases *N*_thermo_ in ConsiGma® 160 is lower than in ConsiGma® 320, indicating an increased energy contribution from the surrounding equipment in ConsiGma® 320.

### Heat Transfer

**Fig. 17:**
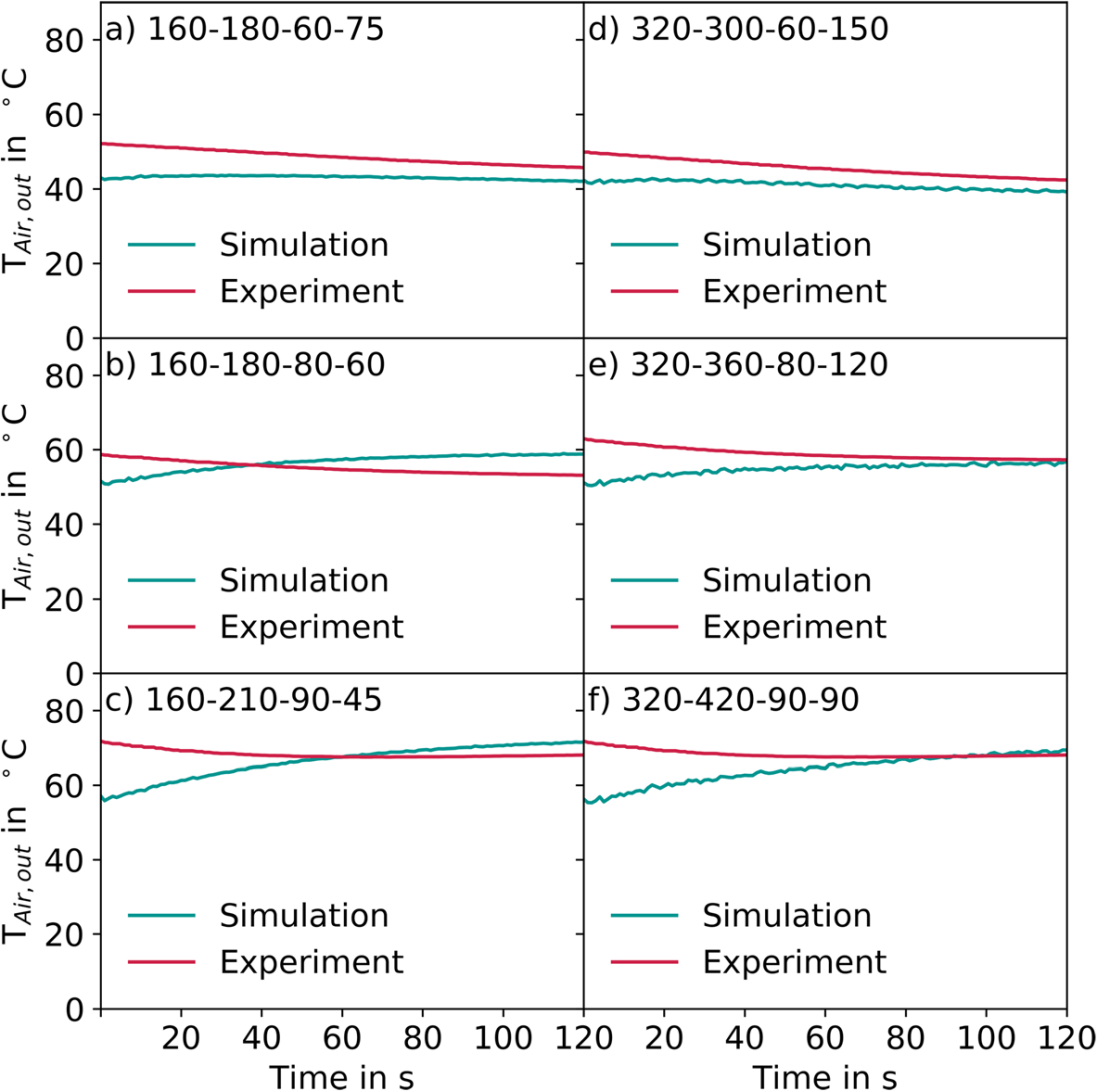
Comparison of the outlet air temperature between experiment and simulation. (**a**) ConsiGma 160, drying air flow rate of 180 m³/h, temperature of 60 °C and spray rate 75 g/min, (**b**) ConsiGma 160, drying air flow rate of 180 m³/h, temperature of 80 °C and spray rate 60 g/min (**c**) ConsiGma 160, drying air flow rate of 210 m³/h, temperature of 90 °C and spray rate 45 g/min (**d**) ConsiGma 320, drying air flow rate of 300 m³/h, temperature of 60 °C and spray rate 150 g/min (**e**) ConsiGma 320, drying air flow rate of 360 m³/h, temperature of 80 °C and spray rate 120 g/min (**f**) ConsiGma 320, drying air flow rate of 420 m³/h, temperature of 90 °C and spray rate 90 g/min

Figure 17 shows the outlet air temperature for all six conditions investigated. On the left- and right-hand sides, the results for ConsiGma® 160 and ConsiGma® 320 are shown, respectively, going from wet to dry conditions. The initial outlet temperatures in the experiments are higher than in the simulated cases. The reason is that the surrounding equipment is heated over time (also in between the coating experiments). The air cools due to the tablets and the coating spray while being heated due to the stored heat of the equipment. In the simulation, the energy input from the equipment and the heat loss to the surrounding air is neglected since only the drum inside the coater housing was considered. As such, the simulated air temperature increases while the experimental one decreases. However, after some time of about 60s the simulations agree very well with the experiments. Under wet conditions, the simulation underpredicts slightly the outlet temperature. It seems that the simulations underestimate the energy input from the equipment and overestimate the evaporation rate. In the moderate and dry cases in ConsiGma® 160, the outlet air temperature is slightly overpredicted by the simulation, which may suggest that the heat loss to the surrounding air in the smaller drum is higher due to longer residence times in the coater housing. In terms of the outlet air temperature, in the moderate and dry cases in ConsiGma® 320 the experimental and simulated results match well. The residence time of the drying air is lower due to an increased air flow rate.

**Fig. 18:**
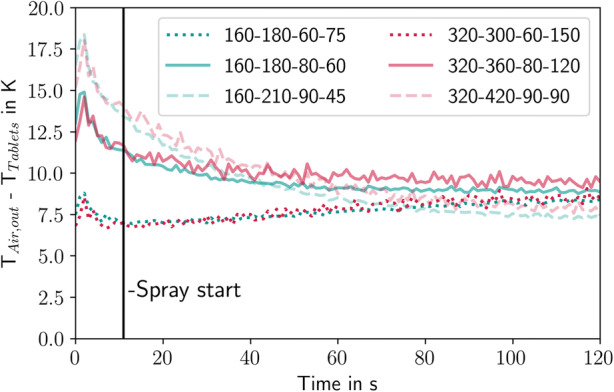
Mean temperature difference between the outlet air and tablet bed in the simulation. The numbers in the plots refer to the coater wheel type, drying air flow rate (m^3^/h), drying air temperature (°C), and spray rate (g/min)

Figure 18 shows the evolution of the difference between the outlet air and tablet temperatures in the simulation. The tablets were initialized with a temperature of 30°C for all cases. After an initial peak at about 10 s, the difference between the air and tablet temperature reaches a steady state. The initial peak indicates the distribution phase (Fig. 4a), with most of the drying air bypassing it. After 5 s, the ring formation begins and the tablets are distributed along the drum wall. This enlarges the air-tablet interaction surface and increases the heat transfer to the tablets. After 12 s, the spray nozzle is activated and the tablet-air temperature difference reaches a steady state after 60 s between 8°C and 10°C. The steady-state does not clearly indicate how the tablet-air temperature difference is influenced by the thermal process settings since the highest temperature corresponds to the medium settings, followed by the wet settings and the dry settings that have the lowest temperature difference.

## CONCLUSION

The goal of this work was to assess the validity of a CFD-DEM simulation model of the ConsiGma® tablet coater. Six experiments were performed in two coater sizes at varying spray rates, drying airflow rates and inlet temperatures. These experiments were repeated *in silico* using coupled CFD-DEM simulations that exchange momentum, heat, and mass between the tablet and the air phase. To validate the tablet bed dynamics, the tablet acceleration during the various process stages was evaluated and compared to the experimental results. Furthermore, the coating mass variability in terms of CoV, mass transfer (evaporation of the coating liquid), and heat transfer (between the gas and the tablet phases) were compared to the experimental results in order to validate the entire coating process.

Overall, the simulation and experimental results were in good agreement. During the ring formation stages, they match well with respect to the tablet acceleration. During the cascade stages, although the spatial- and time-averaged simulation values match well, the simulation overpredicts the acceleration during the free fall. In the simulation the tablets adhered to the wall in this phase, while in the experimental run this phenomenon was not observed possibly due to the difference in the size and shape between the accelerometer and the tablets investigated.

The coating mass variability was tracked in six experiments under constant conditions but with a variable coating mass gain target. In these experiments, the simulation was able to capture the evolution of CoV over time. In the simulation, since the spray drying and overwetting effects were not included, it was impossible to show or predict the coating defects.

The mass and heat transfer were validated by analyzing the outlet air humidity and temperature. The simulations matched the increase in the outlet air humidity during the ramp-up period and at steady-state. Moreover, the simulations were able to keep track of the liquid on the tablets, which can help to explain an increase in the LoD and can be used in future work to predict overwetting of the tablets. In terms of the outlet temperature, the outlet air temperature in the simulation was cooler than it was in the experiments at the beginning. The simulation also showed the temperature difference between the tablets and the outlet air temperature, which was around 10 K in all cases. Since the outlet air temperature and humidity match well, it is assumed that this value is also valid for the ConsiGma® tablet coater.

The simulation model was able to replicate the movement and thermal behavior during the coating process inside the ConsiGma® tablet coater on various scales and in various process settings. The differences between the experimental and model results are small. This validates our simulation approach and confirms that the proposed model can be used to investigate the process space of the ConsiGma® coating process. Furthermore, it can be applied to expand the design space of the ConsiGma® coater and reduce the total number of experiments during the development by excluding process settings that are predicted to fail.

Even though the model captured the main features of the coating process inside the ConsiGma® tablet coater, it can be improved. Three important issues will be addressed in the future: The first one is the spray drying and evaporation of the liquid before a droplet is deposited on the tablet. This model extension will offer a more accurate prediction of the liquid deposition efficiency and identify drier and wetter runs even more accurately. The second one is the liquid absorption deposited from the surface and the tablet drying by diffusion. The third one is the model equipment to account for the influence of heat transfer from the equipment to the tablets and drying air.

## Electronic Supplementary Material

ESM 1 (PNG 256 kb)

High Resolution (TIF 68906 kb)

ESM 2 (DOCX 13 kb)
