# Supplementary figures and images for: Validating a Numerical Simulation of the ConsiGma(R) Coater
Source: AAPS PharmSciTech. 2020 Nov 26;22(1):10. doi: 10.1208/s12249-020-01841-7 (PMC7691303; doi:10.1208/s12249-020-01841-7)

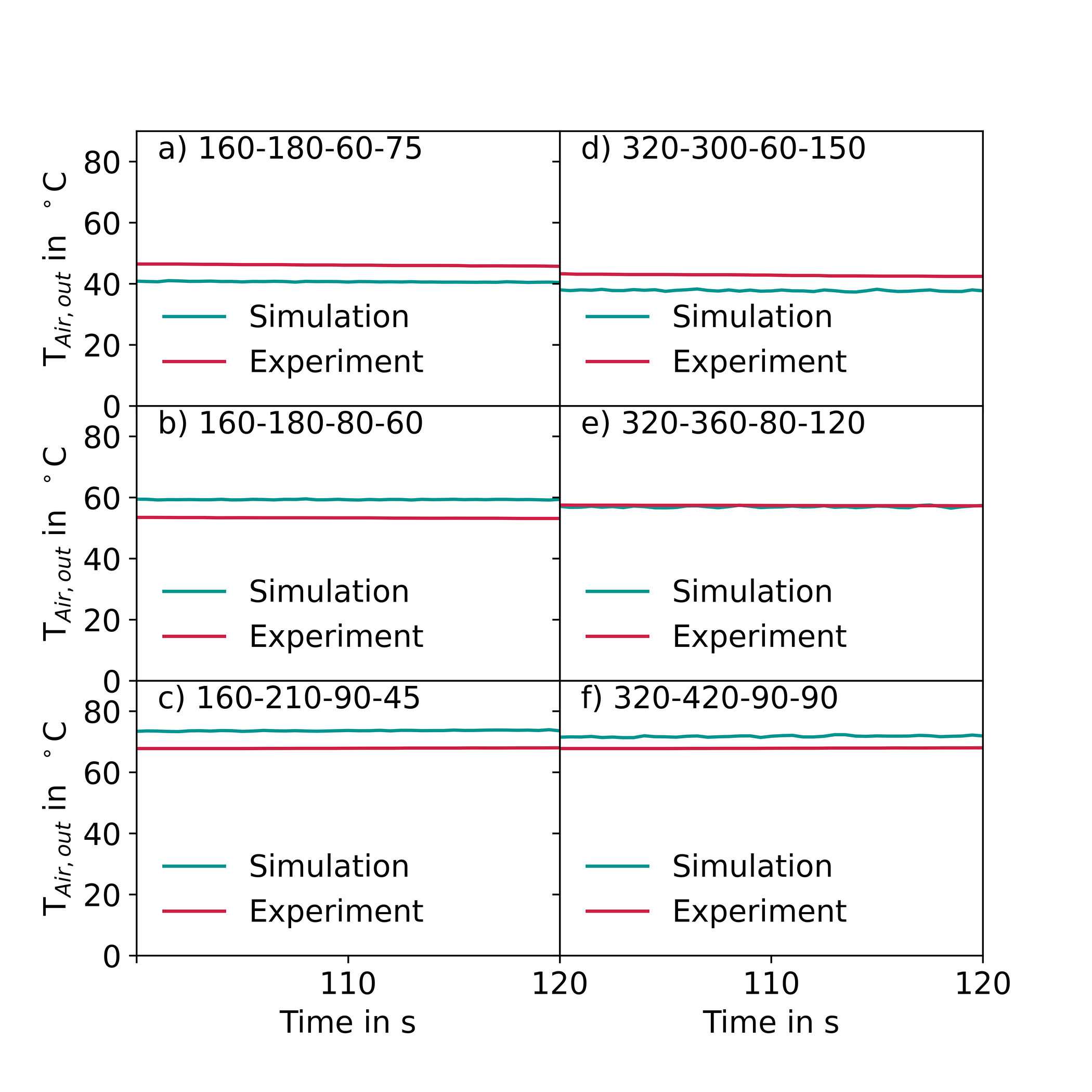

Supplement: Supplementary file 1 — (PNG 256 kb) [file 12249_2020_1841_Fig19_ESM.png]
