# Supplementary material for: Validating a Numerical Simulation of the ConsiGma(R) Coater
Source: AAPS PharmSciTech. 2020 Nov 26;22(1):10. doi: 10.1208/s12249-020-01841-7 (PMC7691303; doi:10.1208/s12249-020-01841-7)
Supplement: Supplementary file 3 — (DOCX 13 kb) [file 12249_2020_1841_MOESM2_ESM.docx]

| Particle properties |  |
| --- | --- |
| Contact stiffness | 10000 N/m |
| Particle density | 1485 kg/m^3^ |
| Heat capacity | 1100 kJ/kg K |
| Interaction Tablet – Wall |  |
| Coefficient of Restitution | 0.5 |
| Static friction coefficient | 0.45 |
| Dynamic friction coefficient | 0.30 |
| Tangential / Normal stiffness ratio | 0.66 |
| Interaction Tablet – Tablet |  |
| Coefficient of Restitution | 0.7 |
| Static friction coefficient | 0.45 |
| Dynamic friction coefficient | 0.30 |
| Tangential / Normal stiffness ratio | 0.66 |
| Simulation |  |
| Time step length | 2·10^-5^ s |
